# Finite element method analysis of the periodontal ligament in mandibular canine movement with transparent tooth correction treatment

**DOI:** 10.1186/s12903-015-0091-x

**Published:** 2015-09-04

**Authors:** Yongqing Cai, Xiaoxiang Yang, Bingwei He, Jun Yao

**Affiliations:** Department of Chemical Engineering, Fuzhou University, Fuzhou, Fujian China; Department of Mechanical Engineering, Fuzhou University, Fuzhou, Fujian China; Department of Orthodontic, Affiliated Hospital of Stomatology, Fujian Medical University, Fuzhou, Fujian China

## Abstract

**Background:**

This study used the 3D finite element method to investigate canine’s displacements and stresses in the canine’s periodontal ligament (PDL) during canine’s translation, inclination, and rotation with transparent tooth correction treatment.

**Methods:**

Finite element models were developed to simulate dynamic orthodontic treatments of the translation, inclination, and rotation of the left mandibular canine with transparent tooth correction system. Piecewise static simulations were performed to replicate the dynamic process of orthodontic treatments. The distribution and change trends of canine’s displacements and stresses in the canine’s PDL during the three types of tooth movements were obtained.

**Results:**

Maximum displacements were observed at the crown and middle part in the translation case, at the crown in the inclination case, and at the crown and root part in the rotation case. The relative maximum von Mises and principal stresses were mainly found at the cervix of the PDL in the translation and inclination cases. In the translation case, tensile stress was mainly observed on the mesial and distal surfaces near the lingual side and compressive stress was located at the bottom of the labial surface. In the inclination case, tensile stress was mainly observed at the labial cervix and lingual apex and compressive stress was located at the lingual cervix and labial apex. In the rotation case, von Mises stress was mainly located at the cervix and inside the lingual surface, tensile stress was located on the distal surface, and compressive stress was detected on the mesial surface. The stress and displacement value rapidly decreased in the first few steps and then reached a plateau.

**Conclusions:**

Canine’s movement type significantly influences the distribution of canine’s displacement and stresses in the canine’s PDL. Changes in canine’s displacement and stresses in the canine’s PDL were exponential in transparent tooth correction treatment.

## Background

The primary aim of orthodontics is to obtain the proper position of the teeth in the dental arch to get the correct occlusion with the best functional and aesthetic features.

Since its advent in 1999, the transparent tooth correction system has become an accepted treatment choice for clinicians. This system is based on clear sequential appliances (aligners) made of a translucent thermoplastic material using computer-aided scanning and imaging techniques [1]. Therefore transparent tooth correction system has its own peculiar biomechanics distinct from that of conventional orthodontics. The orthodontic forces of transparent tooth correction technology mainly result from rebound force of the aligner’s elastic deformation.

Since it is a relatively new method, some aspects are still insufficiently investigated. Previous studies about transparent tooth correction have predominantly concentrated on individual case reports [2–7] or technical or material-specific aspects [8–13], or they addressed oral hygiene [14, 15] and quality of life [15, 16]. However, investigations on the biomechanical questions regarding this technology are few and far between [17, 18]. Orthodontic tooth movement has proven to be an extremely complex process involving a succession of physical, biochemical and cellular reactions, leading to bone remodeling [19]. Apart from the biochemical processes during bone remodeling, the biomechanics of tooth movement is an important topic in orthodontic research [20]. One of particular interests for orthodontists in this field of engineering is the calculation of stresses developed on the tooth and surrounding tissues during orthodontic tooth movement. Other studies have focused on investigating the stresses within the PDL induced by orthodontic forces [21–26]. Too much high stress would cause necrosis of PDL and that may slow the rate of tooth movement.

The finite element (FE) method is used to understand the biomechanics of orthodontic devices because it allows the estimation of stresses, strains, and deformations in different tissue structures, such as alveolar bone, periodontal ligament (PDL), and teeth, during treatment [27–30]. Several studies have employed FE on orthodontic mechanics [31–35].

The majority of FE studies on orthodontic tooth movement have focused on static evaluation of initial loading status, whereas dynamic long-term FE analysis has been rarely performed. Orthodontic tooth movement is not a one-step process, and alterations in the mechanical responses of tissues occur when the tooth is mechanically simulated during orthodontic tooth movement.

This study aimed to (1) simulate the dynamic process of the translation, inclination, and rotation of mandibular canine with transparent tooth correction treatment by using piecewise static 3D FE method and (2) study the distribution patterns and change trends of canine’s displacement and stresses in the canine’s PDL during tooth movement.

## Methods

### Generation of finite element model

#### Modeling of 3D models

**Fig. 1:**
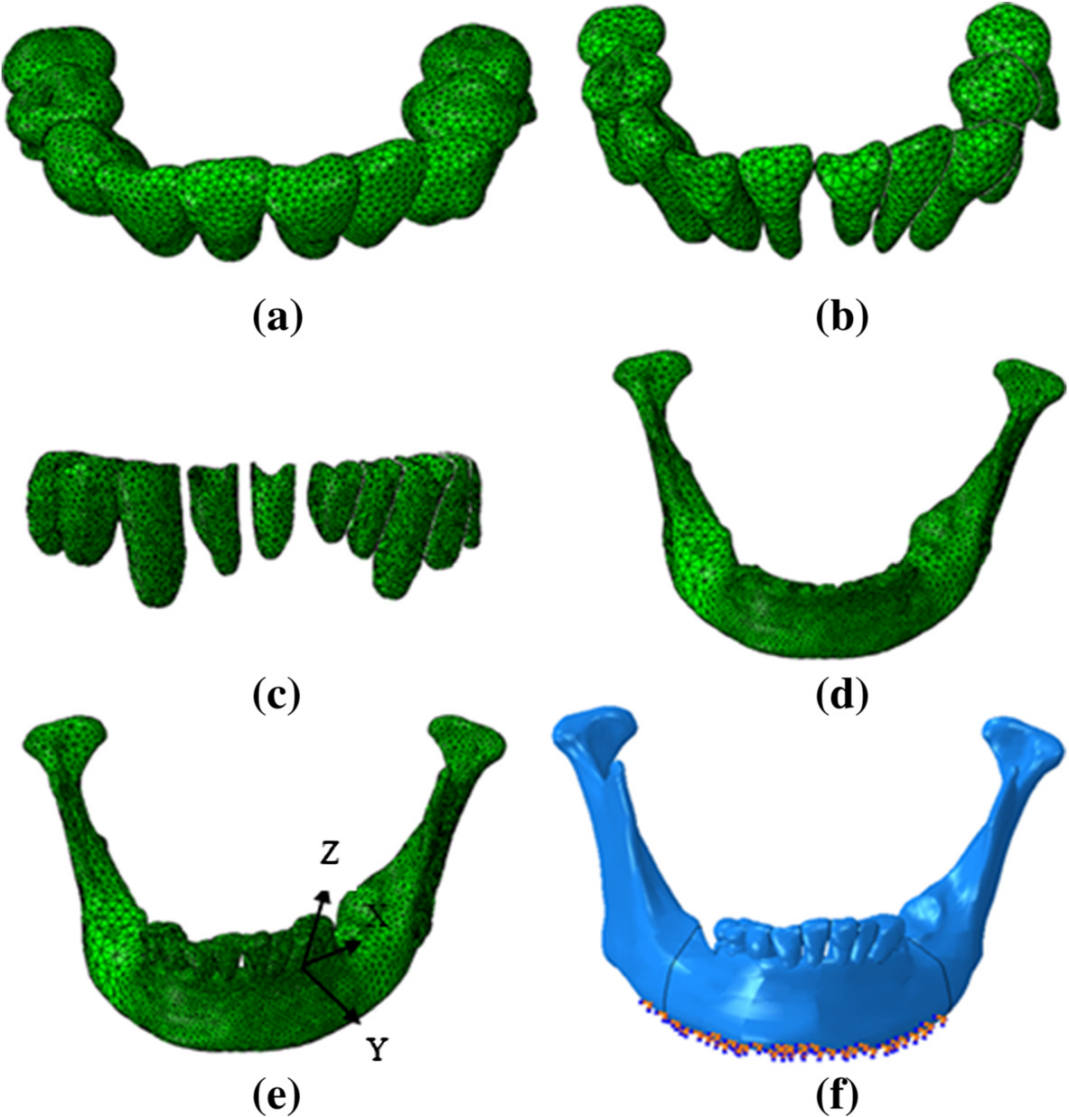
Finite element model of mandibular tissue, Aligner (**a**), Dentition (**b**), periodontal ligament (**c**), mandible (**d**), the assemble model (**e**), load and boundary condition (**f**)

The FE models of mandibular tissues established in our previous investigations were used in the current study [30]. The 3D FE models (Fig. 1), which comprise mandibular anterior teeth, PDL, and alveolar bone, were developed according to the sequential computed tomography (CT, Philips/Brilliance64) images (0.5 mm intervals) of the normal craniofacial of one volunteer. The geometry of the mandible and dental models were reconstructed with the MIMICS (Materialise) and Geomagic Studio (Geomagic) software. The teeth were translationally moved slightly by using the 3-matic (Materialise) software to eliminate contact between the teeth. The 0.25 mm-thick layers around the tooth root were created to represent the PDL, as indicated in previous studies [30, 36–38]. Finally, the constructed models were imported to the FE software ABAQUS for further analysis.

The left mandibular canine (number 33) was selected as the treated tooth. A local coordinate system was created as shown in Fig. 1(e) to apply and measure canine’s movements. The origin was located in the interface of the crown and the root. The coordinate Z axis was coincident with the canine long axis. The Y axis was located in the labial–lingual direction, and the X axis was positioned in the mesial–distal direction. Three kinds of tooth movement were investigated: 0.25 mm translation in the negative direction of the Y axis (from the labial side to the lingual side), 2° rotation (around the canine long axis, the distal part moves from the labial side to the lingual side), and 2° inclination along the X axis (the crown moves from the lingual side to the labial side). The amounts of loads induced by the aligner were determined using the amount of displacement cooperated in the aligner.

The aligner thickness was assumed to be 0.8 mm, and the orthodontic aligner modeling process was as follows [36, 39]:

1. Obtaining the post-treatment dentition-PDL-mandible models. The canine moved to the desired position of the case using the 3-matic software.
2. Thickening of crowns. The crowns of the model obtained in Step 1 were thickened by 0.8 mm in the normal direction of the crowns by using the Geomagic Studio.
3. Merging the thickened crowns. The obtained thickened crowns were imported into ABAQUS and merged as a whole (Boolean add operation).
4. Subtracting the post-treatment models from the merged thickened crown models. The corresponding post-treatment dentition-PDL-mandible model (Obtained in Step 1) was subtracted (Boolean operation) from the model obtained in step 3 to obtain aligner models.

**Fig. 2:**
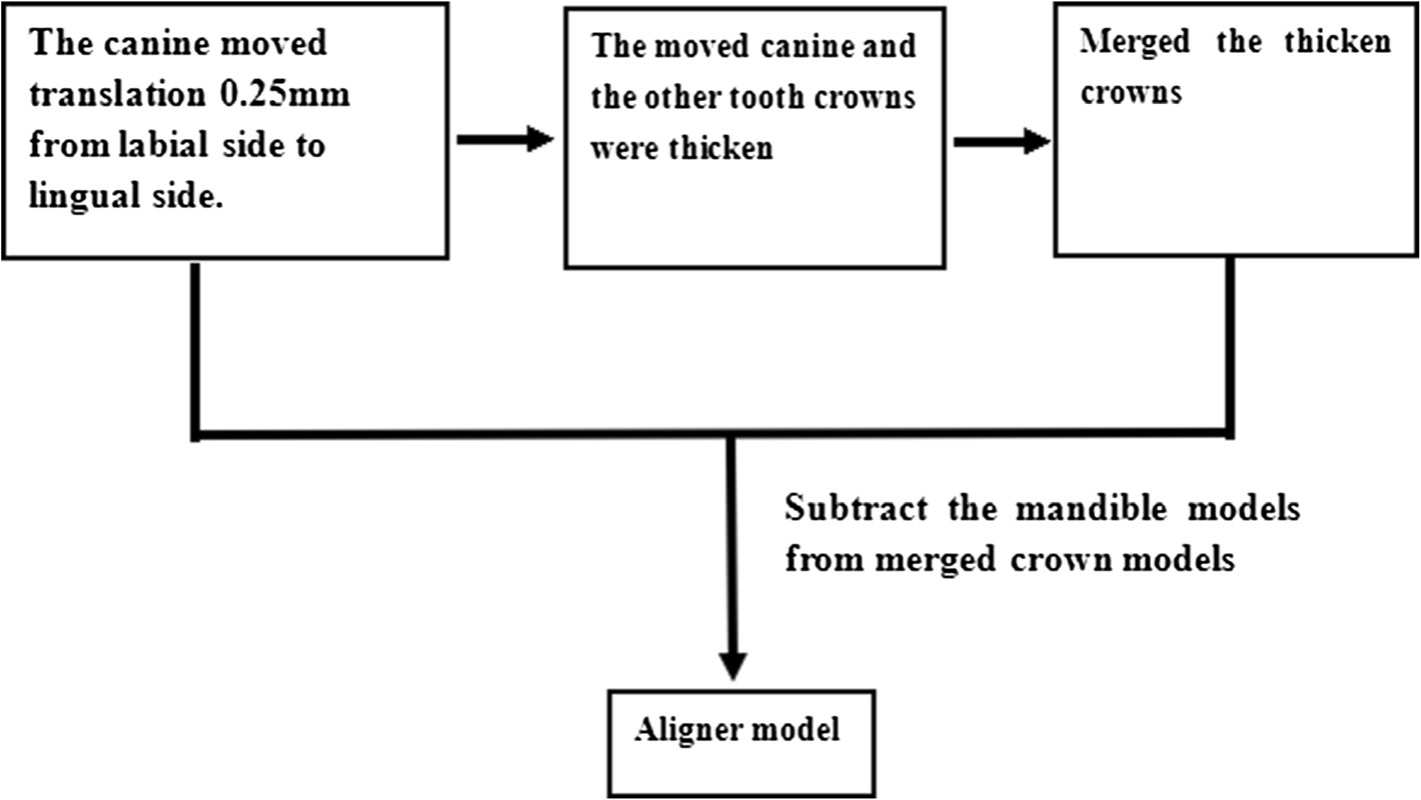
The modeling process of aligner in translation case

Figure 2 describes the four steps of the modeling process, with the translation case as the example.

### Material properties

The mechanical properties of the tooth, PDL, and alveolar bone were assumed to be linear elastic, homogeneous, and isotropic and defined according to previous studies [36, 39], as shown in Table 1.

**Table 1:** Material properties and Unit and node numbers of FE models

Material	Modulus of Elasticity/MPa	Poisson’s ratio	Number of elements	Number of nodes
Teeth	18600	0.31	15457	26371
Alveolar bone	13700	0.30	51502	80282
periodontal ligament	0.68	0.49	12891	26396
Aligner	816.31	0.30	19256	37024

Ten-node tetrahedral element was adopted in the FE models, and the numbers of the elements and nodes for each component of the model are presented in Table 1. The elements were examined with Mesh Verify command in ABAQUS to ensure convergence of the FE model.

### Loading and boundary conditions

The interaction of the crowns and aligner were assumed to be frictionless and every tooth did not come in contact with their adjacent teeth. The bottom and posterior surfaces of the mandible were also fixed. Approximately 5000 nodes were restricted as shown in Fig. 1(f).

### Simulation of tooth movement process

**Fig. 3:**
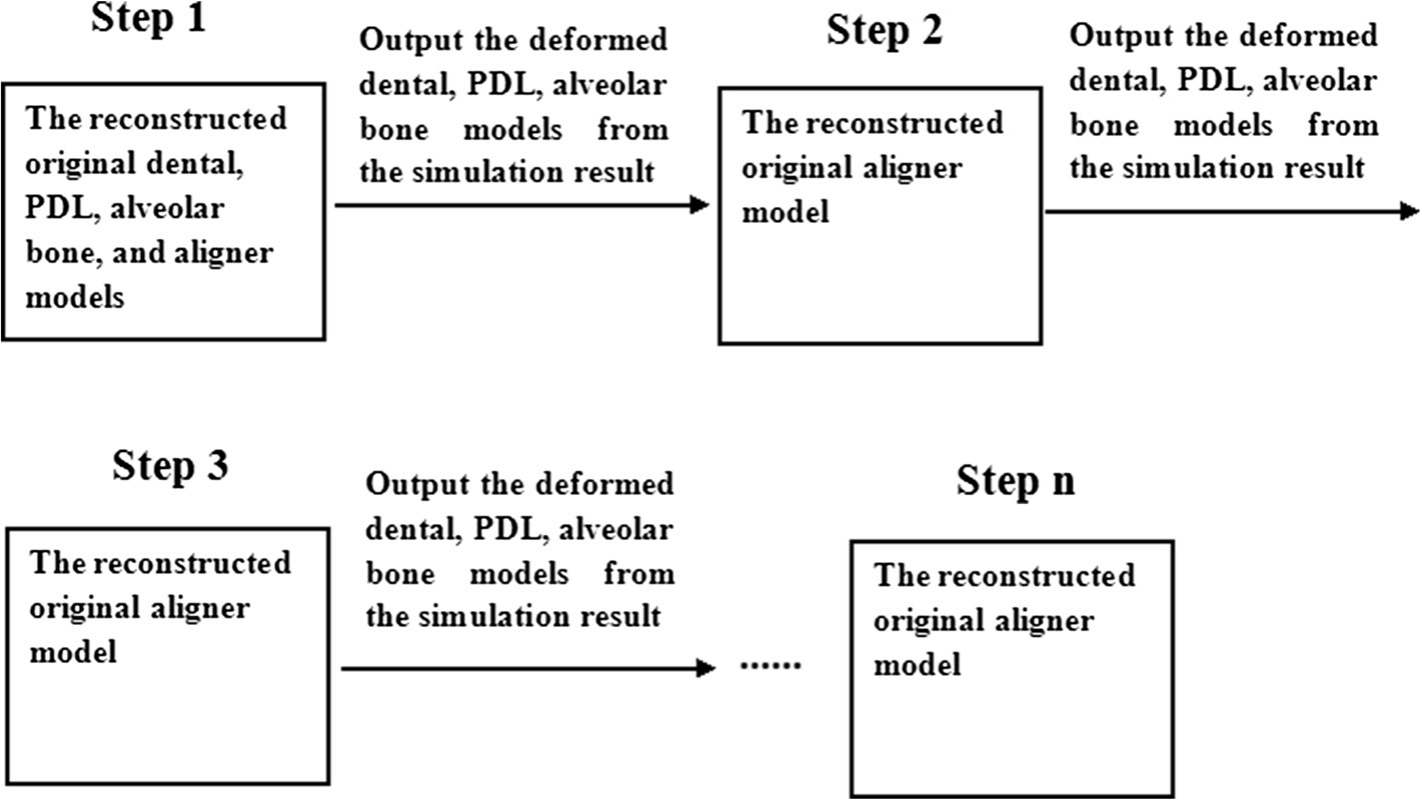
The models used in every step

In this investigation, bone remodeling was assumed to adapt to the tooth deformation and the surrounding structure produced by orthodontic force. Piecewise static simulations were conducted to replicate dynamic orthodontic tooth movement. One static simulation was operated in one step. The deformed canine, PDL, and alveolar bone in the last step of static simulation were obtained and used as the model for the next static simulation. The models used in each step are presented in Fig. 3.

## Results

During simulation, the translation case comprised 61 steps, the inclination case had 15 steps, and the rotation case had 16 steps.

### Canine’s initial displacement

The canine’s displacement patterns differed in the orthodontic treatment process. Table 2 shows the location change in the maximum and minimum displacements of the canine in each case.

**Table 2:** The change of canine’s maximum and minimum displacement location

Cases		Beginning	Later	Final
Translation	Maximum displacement	Crown apical	Crown apical	Middle part
	Minimum displacement	Root part	Crown’s cervix & Root’s apical part	Crown part
Inclination	Maximum displacement	Crown part	Crown part	Crown part
	Minimum displacement	Root part	Crown part	Crown part
Rotation	Maximum displacement	Crown part	Crown & Middle part	Crown & Middle part
	Minimum displacement	Crown apical & Root apex	Root distal side & crown apical	Root distal side & crown apical

**Fig. 4:**
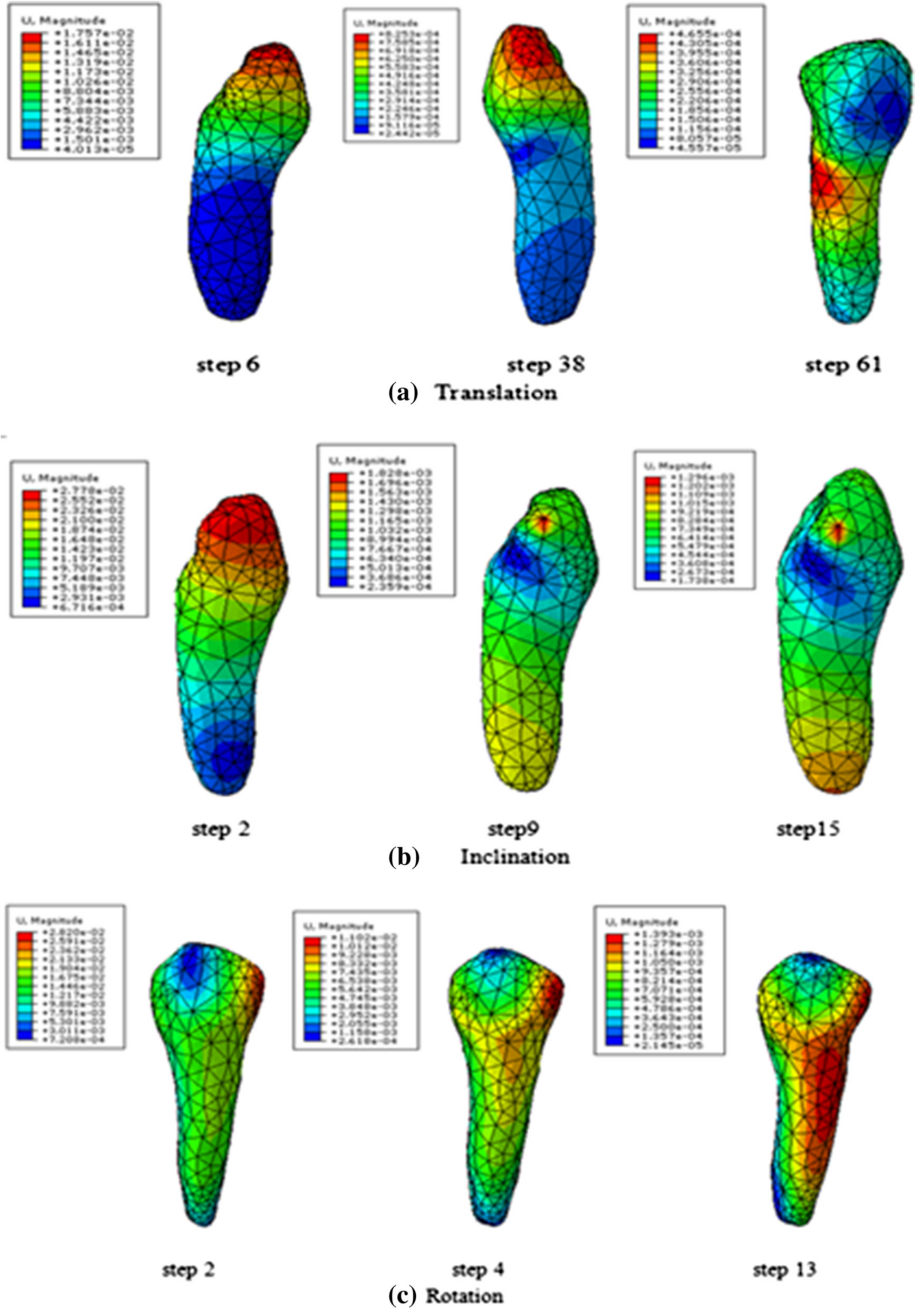
Displacement distribution trend in canine at the beginning, later, and final steps

**Fig. 5:**
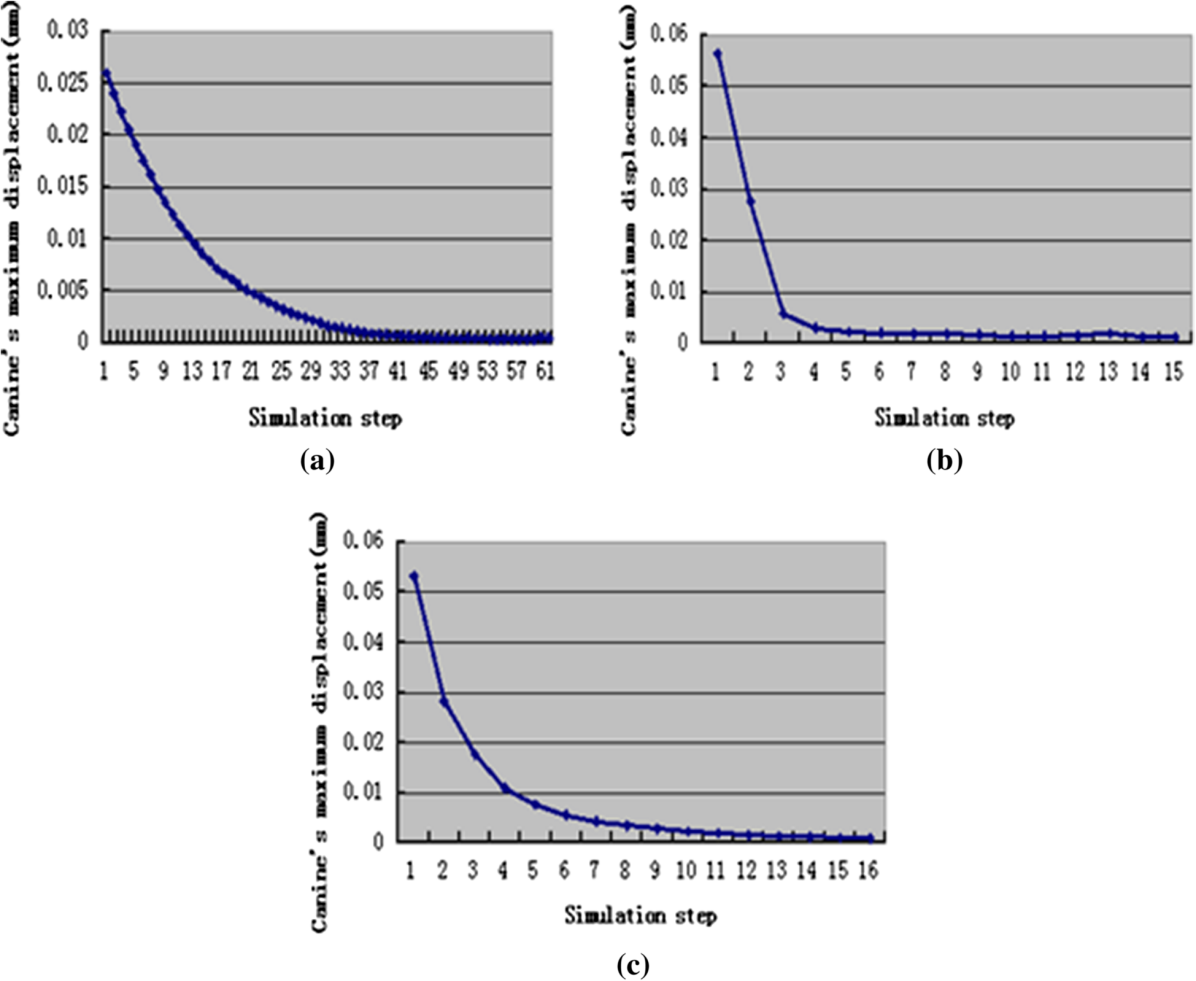
The change trend of canine’s maximum displacement during orthodontic treatment, translation case (**a**), inclination case (**b**), rotation case (**c**)

Figure 4 describes the displacement distribution patterns at the beginning, middle, and final steps. Figure 5 illustrates change trends in the canine’s maximum displacement during orthodontic movement in the three movement cases.

### Stresses of the periodontal ligament

**Fig. 6:**
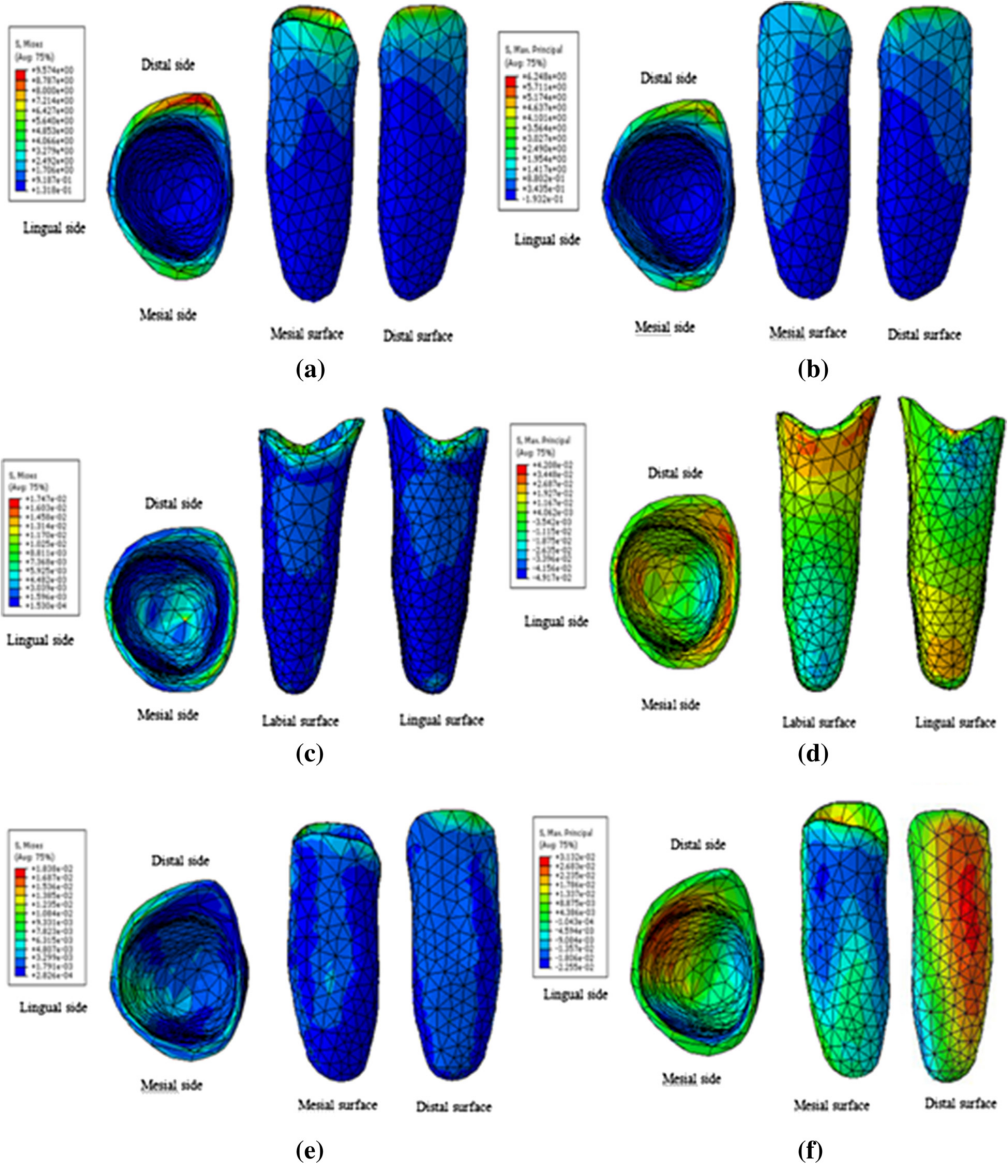
Stresses distribution patterns in PDL, translation von-Mises stress (**a**), translation 1st Principal stress (**b**), inclination von-Mises stress (**c**), inclination 1st Principal stress (**d**), rotation von-Mises stress (**e**), rotation 1st Principal stress (**f**)

Figure 6 explains the stress distribution patterns in the three movement types. As the stress distribution trends were similar throughout the tooth movement process, only the distribution patterns of one step were displayed. The patterns in the three principal stresses were similar; therefore, only the first principle stress was presented.

In the translation case, the highest stresses (von Mises, tensile, and compressive) in canine’s PDL was concentrated at the cervix during tooth movement. Tensile stress was mainly observed on the mesial and distal surfaces near the lingual side, and compressive stress was located at the bottom of the labial surface.

In the inclination case, the highest von Mises stress was concentrated at the cervix and apex. Tensile stress mainly concentrated at the labial cervix and lingual apex, and compressive stress was observed at the lingual cervix and labial apex.

In the rotation case, the highest von Mises stress was mainly found at the cervix and inside the lingual surface. Tensile stress was mainly located on the distal surface, and compressive stress was observed on the mesial surface.

**Fig. 7:**
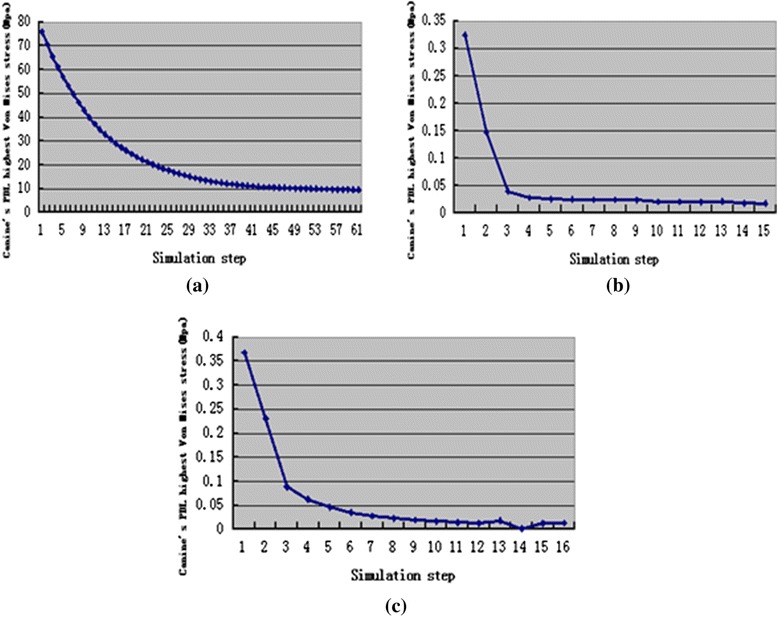
The change trend of canine’s PDL highest von-Mises stress during orthodontic treatment, translation case (**a**), inclination case (**b**), rotation case (**c**)

**Fig. 8:**
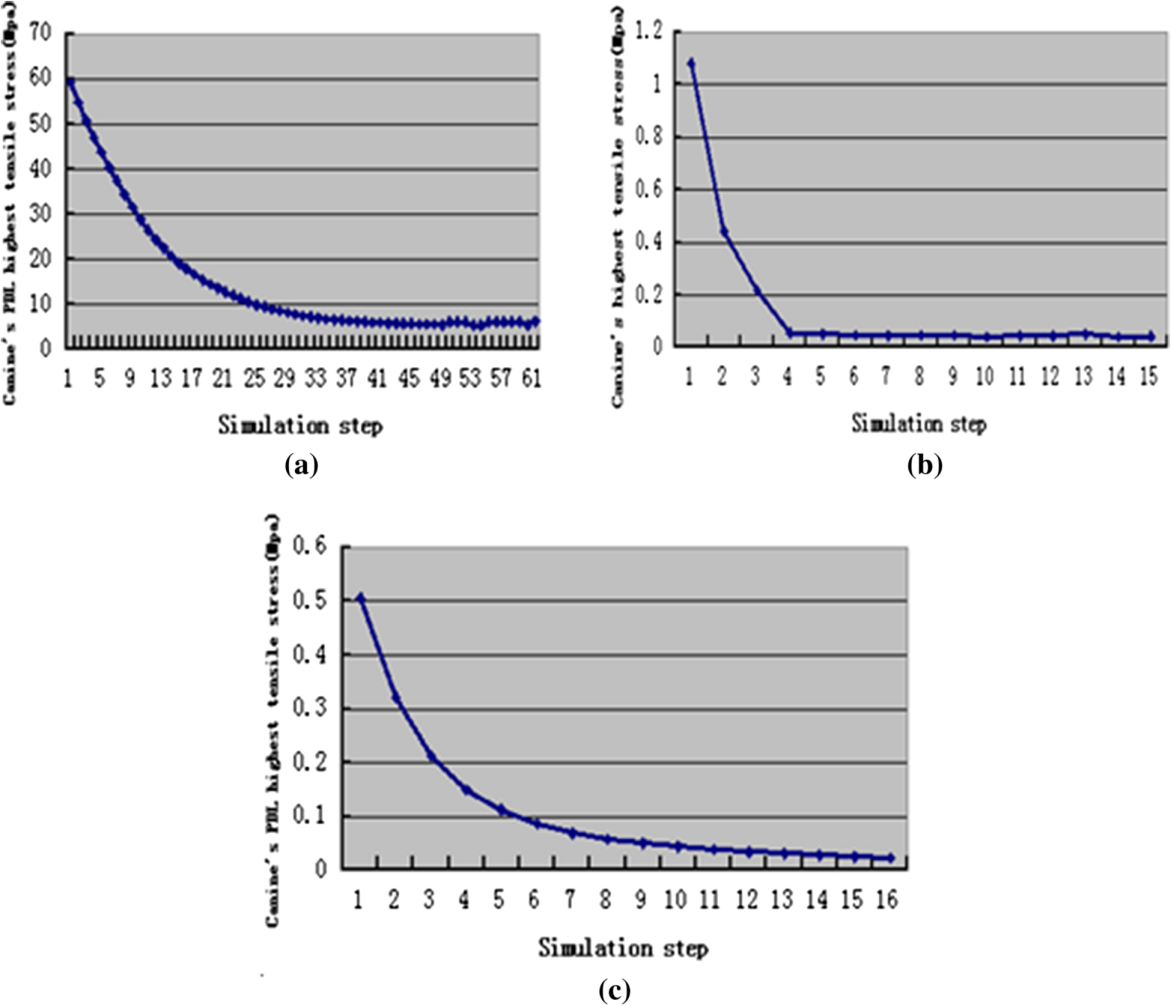
The change trend of canine’s PDL highest tensile stress during orthodontic treatment, translation case (**a**), inclination case (**b**), rotation case (**c**)

**Fig. 9:**
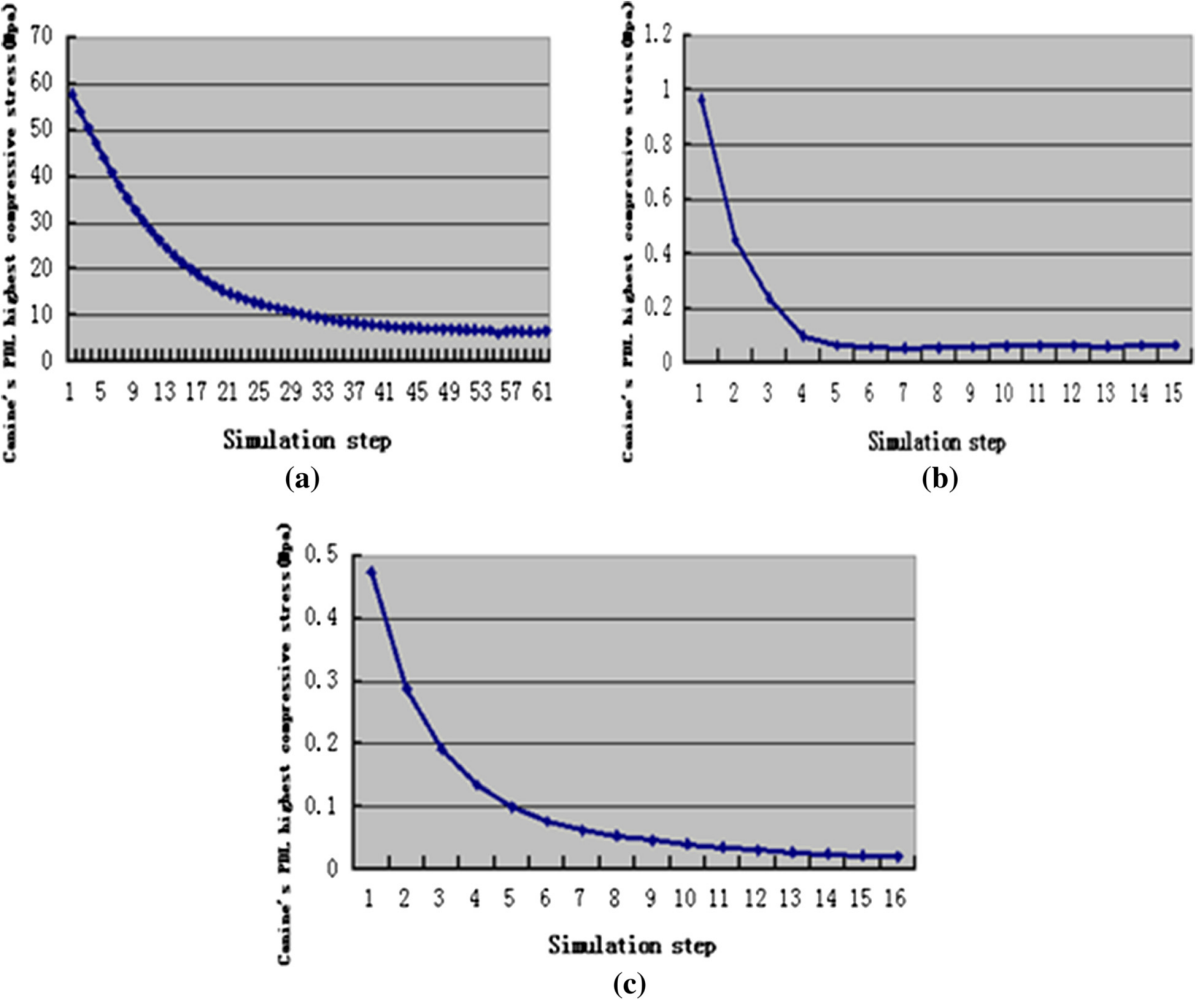
The change trend of canine’s PDL highest compressive stress during orthodontic treatment, translation case (**a**), inclination case (**b**), rotation case (**c**)

Figure 7, Fig. 8, and Fig. 9 present change trends in stresses in the three cases during orthodontic tooth movement.

## Discussion

There are just a few reports about the dynamic simulation of orthodontic tooth movement process [33, 40]. Jing Y et al. [33] and Y. Qian et al. [40] took the normal stress and strain as the bone remodeling stimulus factors, however, neglected the effect of shear stress and strain on bone remodeling. Moreover their simulations need to apply load directly on the tooth, and need to consider the decreased of orthodontic loads.

However, bone remodeling is the activity that bone shows the ability to adapt to a change in external loads, i.e. bone has an optimal structure in the case of mechanical equilibrium, and is capable of remodeling under a changed load until an optimal configuration adapted to the new state of equilibrium is achieved [41, 42]. The present investigation was operated based on this principle.

Calculating the center of rotation (Crot) of the tooth may evaluate the effect of the force system on the tooth movement. The distribution patterns of displacement were different through the process of tooth movement, which indicated vary of rotation center during the treatment process with transparent tooth correction system.

The location of minimum displacement is the approximate location of rotation center.

The rotation center for translation case was located at the root at the beginning and later moved to the middle part and then crown part. The rotation center for inclination case moved from root to crown part. That demonstrated the canine’s translation and inclination were achieved by piecewise inclination movement, the crown part moved first and then the root part moved.

In rotation case, the canine rotated along the canine’s long axis at the beginning, however, with the canine’s movement the canine’s rotation axis deviated from the long axis.

The maximum stress (von Mises, tensile, and compressive) amount during the tooth movement was observed at the first step in the simulations. The highest stress was 75.93 Mpa for the translation case, followed by 1.08 Mpa for the inclination case, and 0.5051 Mpa for the rotation case. The stress in the translation case was higher than the optimal stress of 0.0185 Mpa [43]. This result may be attributed to the translation displacement designed in this study, which was 0.25 mm larger and thus cannot operate an appropriate translation movement. However, in the inclination and rotation cases, the highest stress was slightly higher than the appropriate stress but this high stress rapidly decreased during tooth movement and reached a plateau in a relatively appropriate stress range [21–26].

The distribution patterns of stresses in the PDL are similar throughout the tooth movement process in each case. However, the distribution of stresses and displacements were mainly determined by the canine’s movement types. The maximum displacement for the translation and inclination cases was mainly found at the crown, whereas the minimum displacement at the root and crown. The maximum displacement for the rotation case was located at the crown and middle part. For the canine rotated along the long axis, the minimum displacement was found at the apical of the crown and root.

The highest von Mises stresses for the translation and inclination cases were found at the cervix of PDL. The highest von Mises stress for the rotation case was located in the cervix to the apical of PDL.

Tensile stress for the translation case was mainly observed on the mesial and distal surfaces near the lingual side, and compressive stress was located at the bottom of the labial surface. Tensile stress for the inclination case was mainly found at the labial cervix and lingual apex, and compressive stress was observed at the lingual cervix and labial apex. Tensile stress for the rotation case was observed on the distal surface, and compressive stress was located on the mesial surface.

Changes of canine’s maximum displacement and highest stresses in canine’s PDL during orthodontic tooth movement process were all exponential. That means the change of orthodontic force during orthodontic tooth movement may be exponential in the transparent tooth correction system. This finding is consistent with the experiment result of Simon et al. [18].

According to the simulation results, the orthodontic tooth movement and orthodontic force in transparent tooth correction system can be divided into two stages. In the first stage, the tooth movement and orthodontic force were the maximum at the beginning and then decreased quickly. In the second stage, the tooth movement and orthodontic force kept invariant.

Limitations of this study involve the approximation of the material behavior of the tooth model. The stress–strain relationship was assumed to be linear, elastic, and isotropic. Anisotropic and viscoelastic behavior of the periodontal ligaments was excluded from this model. Some work seems to suggest that this assumption in particular is weak [44]. Secondly, no differentiation was made between cellular and acellular cementum.

## Conclusion

The canine’s movement type had a great influence on distribution of canine’s displacement and stresses in canine’s PDL. Changes of canine’s displacement and stresses in canine’s PDL were exponential during orthodontic tooth movement in transparent tooth correction system.

## Abbreviations

FEM: Finite element method
PDL: Periodontal ligament
FE: Finite element
3D: three-dimensional
CT: Computed tomography
CRot: Center of rotation
